# Knowledge, beliefs and experience of adopting healthy habits in pregnancy: a mixed methods study

**DOI:** 10.1093/eurpub/ckac130.245

**Published:** 2022-10-25

**Authors:** O Kolokotroni, M Economou, A Koutrouba, M Prokou, A Derlagen, P Enayati Zadeh, E Philippou, D Hileti, A Quattrocchi, MC Mosquera

**Affiliations:** 1 School of Health Sciences, Cyprus University of Technology, Limassol, Cyprus; 2 Medical School, University of Nicosia, Nicosia, Cyprus; 3 Department of Life and Health Sciences, University of Nicosia, Nicosia, Cyprus

## Abstract

**Background:**

Early life exposures affect a child's obesity risk. The EARLY START uses participatory action research to develop an intervention for reducing early life obesogenic exposures. The initial phase uses a mixed methods approach to investigate pregnant mothers’ knowledge, beliefs, and experience of adopting healthy dietary and physical activity (PA) habits.

**Methods:**

Cypriot pregnant women in 2021 completed a web-based questionnaire on: a) Adherence to Mediterranean diet (MD) (MEDAS tool); b) knowledge, beliefs on diet and PA. A subgroup participated in a structured focus group discussion of their experience/needs in adopting healthy habits. Data were analyzed using Descriptive and Thematic Content methods.

**Results:**

Ninety-seven women participated, 73% <35 y.o., 49% primigravida, 92% with tertiary education. Adherence to MD was moderate (median 6/14, IQR 2.5), 90% were eating <3 portions of fruit/vegetables daily, 50% believed their diet was healthy and did not change habits in pregnancy. Most had access to information (94%), internet was the commonest source (74%), and the doctor the most trusted (47%). Mild and moderate-intensity PA were considered appropriate by many (60%) for the first and second half of pregnancy, respectively. Most (90%) were aware of the risks of excessive weight gain in pregnancy. Qualitative analysis showed that women value diet as “the main driver to holistically achieving a healthy pregnancy”. The main barrier was the “struggle between the will and ability”. PA was considered a “therapy” but the challenge was “to achieve the right balance”. Internet was described as “accessible but unreliable information source”. Women believed that needs can be met by “early, holistic recommendation-based interventions run by professionals”.

**Conclusions:**

A huge gap exists between knowledge, beliefs, and practice of healthy behaviours in pregnancy. New interventions should meet gaps and needs using contextualized, timely, holistic, and reliable approaches.

**Key messages:**

- Exploring knowledge and experience of healthy habits in pregnancy as part of participatory action research in shaping an obesity prevention intervention revealed important gaps and unmet needs.
- Future interventions should holistically address knowledge gaps and behavioral change needs using contextualized, timely, holistic and reliable information and methods.

